# Genotypic Effects of Fertilization on Seedling Sweetgum Biomass Allocation, N Uptake, and N Use Efficiency

**DOI:** 10.1100/tsw.2001.275

**Published:** 2001-10-23

**Authors:** Scott X. Chang, Daniel J. Robison

**Affiliations:** Center for Enhanced Forest Management, Department of REnewable Resources, University of Alberta, Edmonton, Canada

**Keywords:** half-sib, genotype, G × E interaction, fertilization, genetic improvement, shoot, root

## Abstract

Screening and selecting tree genotypes that are responsive to N additions and that have high nutrient use efficiencies can provide better genetic material for short-rotation plantation establishment. A pot experiment was conducted to test the hypotheses that (1) sweetgum (Liquidambar styraciflua L.) families have different patterns in biomass production and allocation, N uptake, and N use efficiency (NUE), because of their differences in growth strategies, and (2) sweetgum families that are more responsive to N additions will also have greater nutrient use efficiencies. Seedlings from two half-sib families (F10022 and F10023) that were known to have contrasting responses to fertility and other stress treatments were used for an experiment with two levels of N (0 vs. 100 kg N/ha equivalent) and two levels of P (0 vs. 50 kg P/ha equivalent) in a split-plot design. Sweetgum seedlings responded to N and P treatments rapidly, with increases in both size and biomass production, and those responses were greater with F10023 than with F10022. Growth response to N application was particularly strong. N and P application increased the proportional allocation of biomass to leaves. Under increased N supply, P application increased foliar N concentration and content, as well as total N uptake by the seedlings. However, NUE was decreased by N addition and was higher in F10023 than in F10022 when P was not limiting. A better understanding of genotype by fertility interactions is important in selecting genotypes for specific site conditions and for optimizing nutrient use in forestry production.

## INTRODUCTION

Intensive forest management and short-rotation tree crop production will increasingly rely on genetic improvement to capture site resources and on developing genotypes that are suited to specific sites[1]. In agronomic crop production systems, genotypes are increasingly being selected for specific traits; for example, (1) genotype has been found to have a strong influence on wheat quality and good quality lines are recommended for specific environments[2], (2) genotype × N interaction in wheat was significant for grain yield, aboveground dry matter, and number of kernels per spike[3], and (3) tomato genotypes showed strong differences in utilization and assimilation of foliar $NO_3^-$ and tomato yields[4]. Approaches similar to those used in agronomic crops have been explored in forestry to improve forest site productivity and to efficiently capture site resources[5,6,7,8].

As an important hardwood species in the U.S. southeast[9,10], sweetgum is being developed for short-rotation plantation production for fiber[11]. Sweetgum has a wide natural distribution in the eastern U.S., ranging from Connecticut to central Florida and eastern Texas, and thus exhibits considerable genotypic variation[9]. Understanding the interactions between genotype and environment and their effect on sweetgum growth and site productivity will improve our ability to prescribe silvicultural treatments for specific sites and genotypes. In such a way, potential negative environmental problems may be avoided or minimized if intensive management is practiced.

The objective of this study was to explore the genotype × fertility interaction in sweetgum biomass production, N uptake, and N use efficiency (NUE). An earlier study showed that sweetgum families responded to nutrient additions differently[12]. Two half-sib families were selected from the ca. 350 families in the North Carolina State University – Hardwood Research Cooperative (HRC) genetic improvement program. In an earlier work within the HRC, these two families showed different responses to fertility treatments[13]. We hypothesize that (1) sweetgum families have different patterns in biomass production and allocation, N uptake, and NUE because of their differences in growth strategies, and (2) sweetgum families that are more responsive to N additions will also have greater nutrient use efficiencies. N use efficiency is defined here as the amount of biomass produced per unit of N taken up by sweetgum seedlings.

## MATERIALS AND METHODS

This experiment was conducted in pots (22 cm in diameter, 25 cm deep) outdoors at the Horticulture Field Laboratory of North Carolina State University in Raleigh. Seed from two half-sib sweetgum families (F10022 and F10023) from the North Carolina State-HRC genetic improvement program were collected in a seed production area in St. George, SC (on South Carolina Forestry Commission land). Stratified seeds were sown, two per pot from the same family, on 22 July 1999 into pots containing peat, vermiculite, and field soil in a 6:3:1 (volume) ratio. The field soil (Congoree silt loam) was collected from an area with naturally occurring sweetgum within the Schenck Memorial Forest in Raleigh, NC. All pots received a one-time application of 1.45 g of Osmocote[tm] slow-release fertilizer (14-14-14% of N-P-K) 3 weeks after germination to ensure adequate nutrition for healthy seedling development in the first growing season. Seedlings were thinned to one per pot in September 1999 to leave similarly sized plants among pots. Seedlings were over-wintered outdoors. Pots were widely spaced throughout the experiment to eliminate mutual shading.

During the second growing season (6 July 2000), four treatments were applied to both families: (1) no N or P (control); (2) 100 kg/ha equivalent N (N100); (3) 50 kg/ha equivalent P (P50); and (4) 100 kg/ha equivalent N plus 50 kg/ha equivalent P (N100-P50). The experiment used a randomized split-plot design with N treatment assigned to the main plot level, P treatment to the sub-plot level, and family to the sub-sub-plot level. There were three pots for each family within each sub-sub-plot. Fertilizers were applied as granular $NH_4NO_3$ and triple superphosphate. Pots received daily overhead irrigation in each growing season. Initial seedling size (ground-line diameter, total height) was measured on 23 June 2000, before the treatments were applied. At the same time, foliar samples were collected for total N analysis. Final harvest took place on 20 September 2000.

At final harvest, seedlings were cut at ground-line, brought back to the laboratory, and separated into leaf, branch, and stem components. Roots were gently washed free of the growing media. Root systems were not restricted in their growth by the pot size in any of the treatments, as judged from the relative root system size and pot volume. Samples were dried at 65°C and weighed. Components from the three pots of the same family were combined for each sub-sub-plot and ground for chemical analysis. Samples were analyzed for total N concentration by a dry combustion method (Carlo-Erba model NA-1500) by the North Carolina Department of Agronomy.

Statistical analyses were performed with SAS (SAS Institute, Cary, NC, Version 7.01). Normality of distribution and homogeneity of variance were checked by the PROC UNIVARIATE procedure and also by plotting the residuals against the predicted values. Logarithmic transformation was performed on data sets that were not normally distributed and all data sets were normally distributed after the transformation. However, means are reported on untransformed data. Seedling biomass data were tested with initial seedling size as the covariate and adjusted means are reported if the covariate was significant. N concentrations in samples from the final harvest were also tested for significance of covariance using the initial foliar N concentration as the covariate.

## RESULTS

Total tree biomass at the final harvest was significantly related to initial seedling size (ANOVA not shown), and was significantly affected by both N and P treatments (Fig. 1 and Table 1). N and P application increased overall seedling biomass by 52 and 16%, respectively. Family 10023 showed an advantage over F10022 in producing greater amounts of biomass regardless of the treatment level ($p < 0.05$, Fig. 1 and Table 1). The overall seedling biomass was 11% greater in F10023 than in F10022.

N application increased the proportional production of leaf and branch biomass ($p < 0.05$, Fig. 2 and Table 1). There was a greater proportional leaf biomass production in F10023 than in F10022 ($p < 0.05$). P application also increased the proportional production of branch biomass, regardless of the N level. Proportional stem biomass production was greater in F10022 than in F10023 ($p < 0.05$); however, %stem biomass was not affected by the N or P treatment. In contrast, N and P application decreased proportional root biomass production, resulting in smaller root/shoot ratios ($p < 0.05$, data not shown), but there was no genotype effect on biomass allocation to roots.

N and P treatments significantly affected foliar N concentrations at the end of the 2-month experiment period (Fig. 3a and Table 2). P addition did not increase foliar N concentration when no N was applied, but increased foliar N levels when N was applied. This resulted in a significant N × P interaction for foliar N concentration (Fig. 3a and Table 2). This indicated that under the experimental condition, N was the key limiting factor for sweetgum growth. Family 10022 consistently had higher ($p < 0.05$) foliar N concentrations than F10023 regardless of the N or P treatment level.

N treatment effect on branch %N was significant only at the $\alpha = 0.1$ level, and no P effect was found on branch %N (Fig. 3b and Table 2). As was the case for foliar %N, F10022 consistently

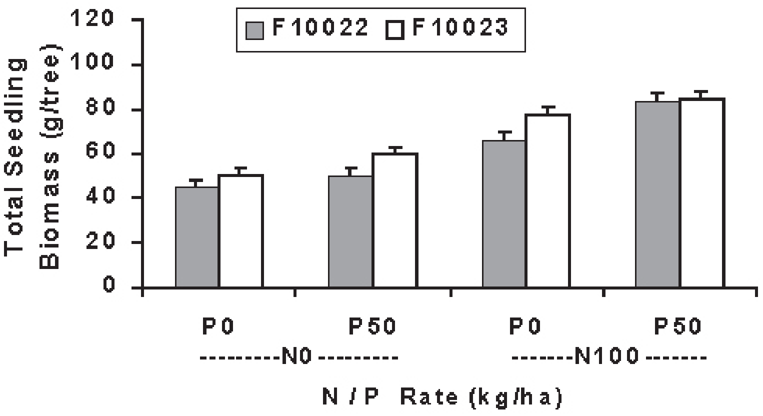

**FIGURE 1.** Effects of N and P application and genotype on total biomass production in a pot study of sweetgum during the second growing season. Vertical bars are standard errors of the means.

**TABLE 1**
**Analysis of Variance (*p* Values) for the Effect of N and P Application, and Genotype on Total Seedling Biomass and Biomass Allocation in Different Components in a Pot Study of Sweetgum during the Second Growing Season**

| | | | % of Biomass Distribution | | | |
|---|---|---|---|---|---|---|
| Variable | df | Total Biomass | Foliage | Branch | Stem | Root |
| N | 1 | 0.056 | 0.005 | <0.001 | 0.688 | 0.005 |
| P | 1 | 0.017 | 0.288 | 0.008 | 0.501 | 0.013 |
| N × P | 1 | 0.435 | 0.864 | 0.910 | 0.090 | 0.117 |
| Family (F) | 1 | 0.007 | 0.034 | 0.337 | <0.001 | 0.860 |
| N × F | 1 | 0.816 | 0.297 | 0.725 | 0.053 | 0.334 |
| P × F | 1 | 0.415 | 0.140 | 0.112 | 0.108 | 0.017 |
| N × P × F | 1 | 0.115 | 0.256 | 0.826 | 0.121 | 0.826 |

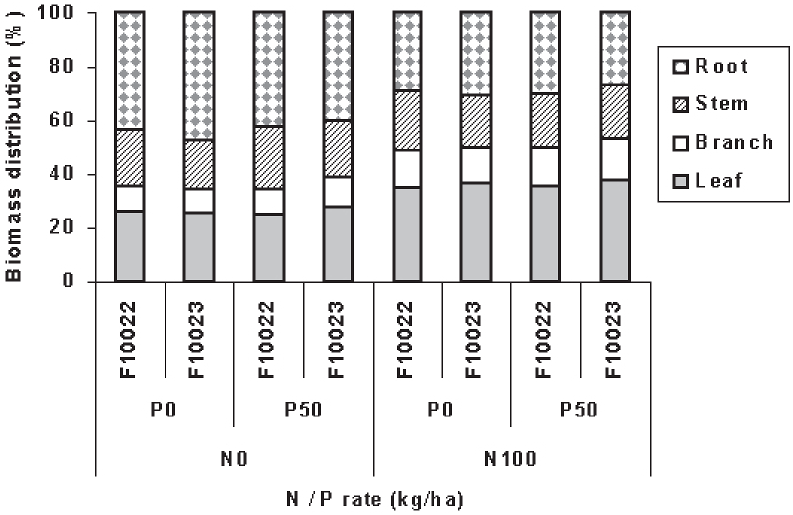

**FIGURE 2.** Effects of N and P application and genotype on biomass distribution in above- and belowground components in a pot study of sweetgum during the second growing season.

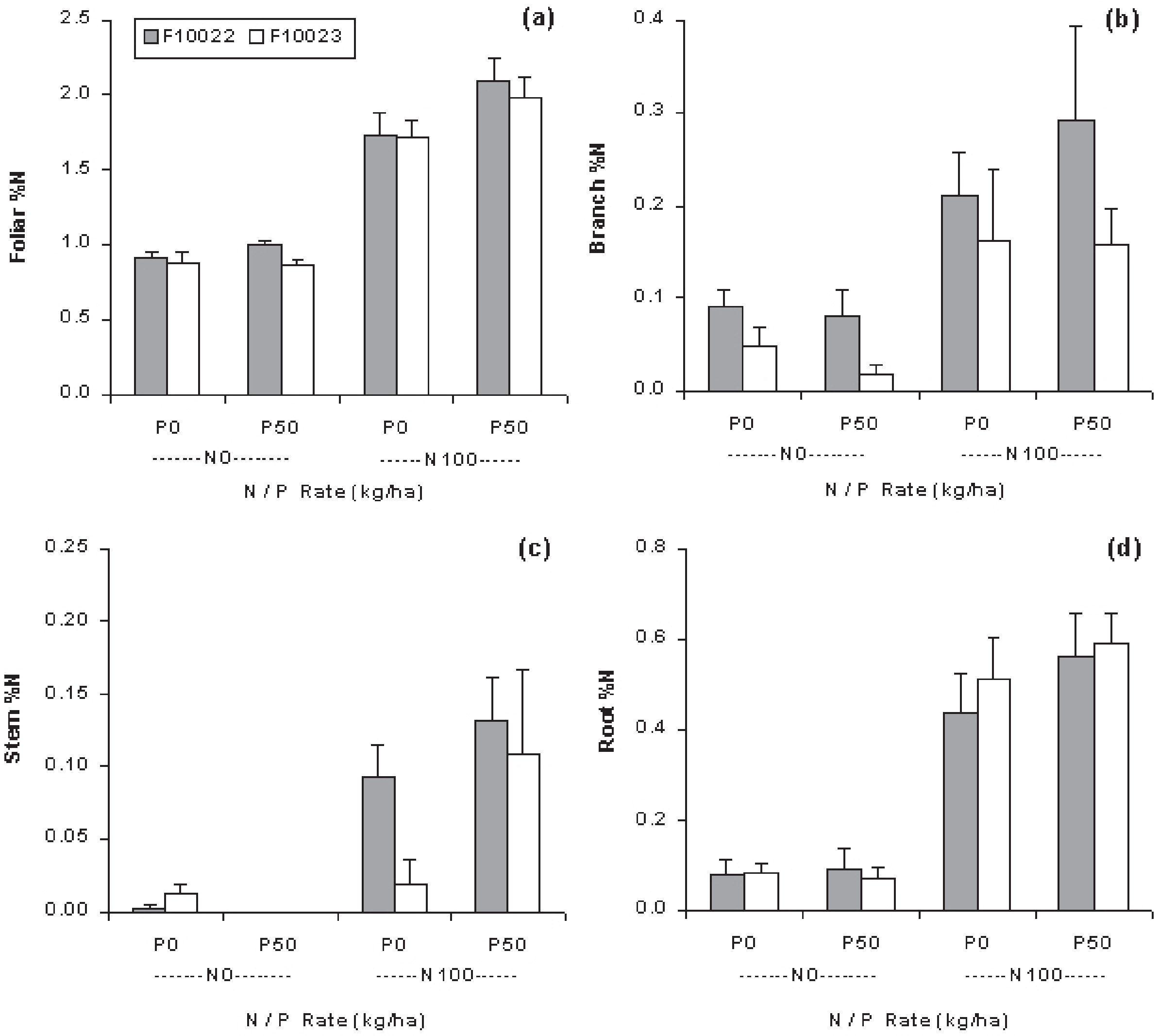

**FIGURE 3.** Effects of N and P application and genotype on N concentration in above- and belowground components in a pot study of sweetgum during the second growing season. Vertical bars are standard errors of the means.

**TABLE 2**
**Analysis of Variance (*p* Values) for the Effect of N and P Application, and Genotype on N Concentration in Sweetgum Foliar, Branch, Stem, and Root Samples Collected at Final Harvest in a Pot Study of Sweetgum during the Second Growing Season**

| | | %N | | | |
|---|---|---|---|---|---|
| **Variable** | **df** | **Foliage** | **Branch** | **Stem** | **Root** |
| N | 1 | 0.003 | 0.085 | 0.047 | 0.008 |
| P | 1 | <0.001 | 0.630 | 0.071 | 0.247 |
| N P | 1 | 0.001 | 0.133 | 0.033 | 0.247 |
| Family (F) | 1 | 0.045 | 0.015 | 0.221 | 0.438 |
| N × F | 1 | 0.898 | 0.442 | 0.139 | 0.309 |
| P × F | 1 | 0.145 | 0.296 | 0.590 | 0.541 |
| N × P × F | 1 | 0.869 | 0.531 | 0.413 | 0.849 |

had higher branch %N than F10023. Both stem and root %N was affected by N addition, but neither P nor family had any significant effect on stem or root %N (Figs. 3c and 3d, Table 2).

The effect of N and P treatments on foliar N content was similar to that of foliar %N, except that foliar N content was seen to be consistently higher in F10023 than in F10022 (Fig. 4a and Table 3). Trends in branch and stem N content followed that of branch and stem N concentration, respectively, except that differences between N treatments (for the branch component) and between P treatments at N100 (for the branch and stem components) became statistically significant (Figs. 4b and 4c, Table 3). Root N content was only affected by the N treatment (Fig. 4d and Table 3).

F10022 consistently had greater foliar N but less branch N content than F10023. Under high N treatment, F10022 accumulated more N in the stem than F10023 (Fig. 4c and Table 3). No family effects other than these were observed for N content in seedling components.

Total N uptake was dramatically increased by N application, and also by P application provided N was added simultaneously (Fig. 5a and Table 3). There was no genotype effect on total seedling N uptake. NUE decreased as N availability increased, regardless of P availability; however, decreased NUE with P application was only observed under low N availability conditions (Fig. 5b and Table 3). A significant P × F interaction revealed that under adequate N and P supply, F10023 had greater NUE than F10022 (Fig. 5b and Table 3); otherwise, NUE was not affected by genotypic differences.

## DISCUSSION

### Biomass Production and Allocation

Biomass production of both half-sib families tested in this experiment was very responsive to N and P additions (Fig. 1). This

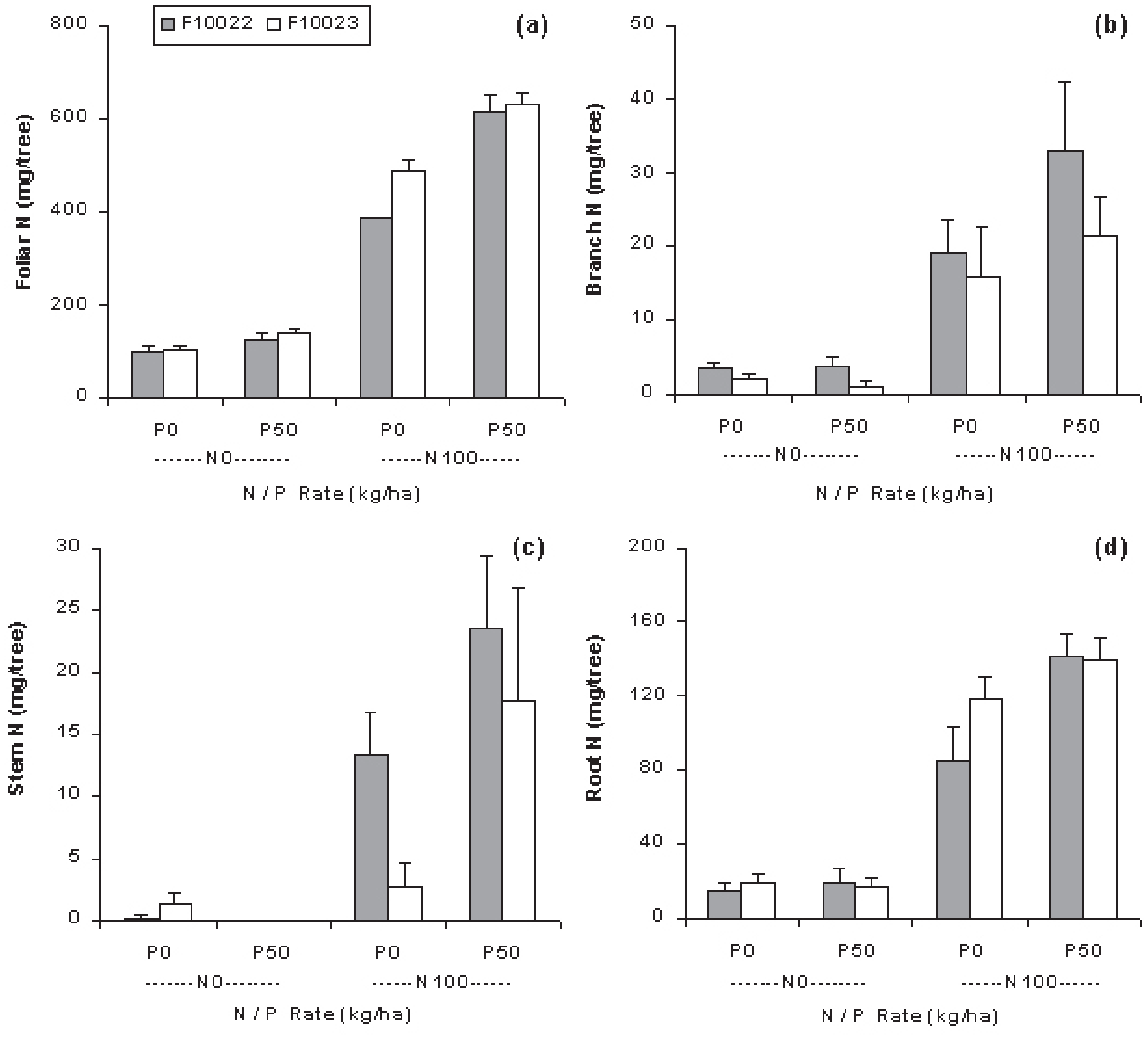

**FIGURE 4.** Effects of N and P application and genotype on N content in above- and belowground components in a pot study of sweetgum during the second growing season. Vertical bars are standard errors of the means.

**TABLE 3**
**Analysis of Variance (*p* Values) for the Effect of N and P Application, and Genotype on N Content in Sweetgum Foliar, Branch, Stem, and Root Samples, Total N Uptake, and NUE in a Pot Study of Sweetgum during the Second Growing Season**

| | | N Content | | | | | |
|---|---|---|---|---|---|---|---|
| Variable | df | Foliage | Branch | Stem | Root | Total | NUE |
| N | 1 | <0.001 | 0.009 | 0.017 | 0.004 | <0.001 | <0.001 |
| P | 1 | <0.001 | 0.405 | 0.020 | 0.788 | <0.001 | 0.008 |
| N × P | 1 | <0.001 | 0.027 | <0.001 | 0.401 | <0.001 | 0.046 |
| Family (F) | 1 | 0.030 | 0.025 | 0.107 | 0.265 | 0.132 | 0.256 |
| N × F | 1 | 0.095 | 0.64 | 0.026 | 0.755 | 0.257 | 0.879 |
| P × F | 1 | 0.210 | 0.726 | 0.722 | 0.558 | 0.166 | 0.044 |
| N × P × F | 1 | 0.106 | 0.725 | 0.266 | 0.746 | 0.150 | 0.311 |

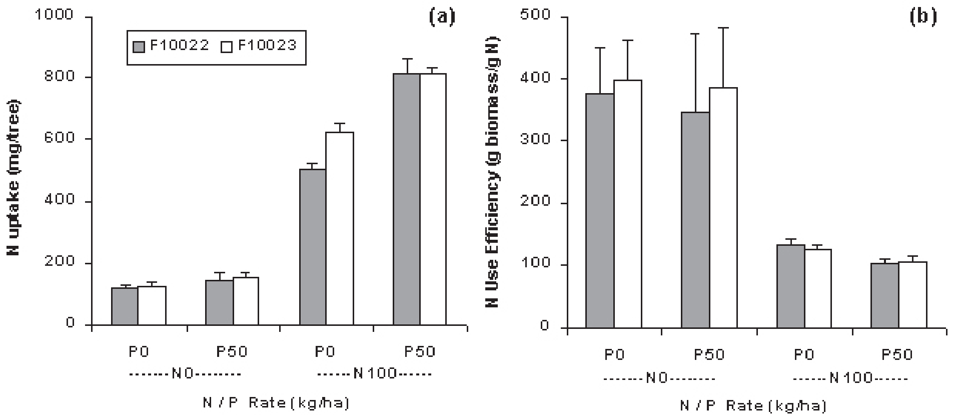

**FIGURE 5.** Effects of N and P application and genotype on N uptake and NUE in a pot study of sweetgum during the second growing season. Vertical bars are standard errors of the means.

is consistent with seedling size measurements reported in Chang and Robison[14] and a number of other studies[12,13,16]. Results from this study indicate that for total seedling biomass production the effect of N and P was additive, as there was no significant N × P interaction for total seedling biomass production. Similar to results in Ku et al.[16], biomass production was much more responsive to N than to P application. Nelson et al.[15] showed that foliar biomass accumulation responded to N application immediately, and, among the rates tested, the lowest rate (100 kg N/ha) was as effective as the other higher rates tested. In this study, the greatest biomass increase over the control was in the pots applied with 100 kg N and 50 kg P/ha. In terms of seedling biomass production, it is clear that maximum production was achieved when both adequate N and P were supplied.

F10023 produced more biomass than F10022 regardless of the N or P treatment. F10023 was more responsive to N and P additions in increasing leaf area[14], and subsequently in leaf and branch biomass (data not shown), two components that can change quickly in response to N and P treatments. This illustrates that for short-rotation sweetgum plantations grown in the U.S. southeast, it is possible to increase fiber production by selecting genotypically superior families. Nelson et al.[15] reported differences in foliar biomass production between two half-sib families in response to N application. This is an indication of the potential for achieving growth gains in sweetgum by selecting responsive genotypes.

Plants are known to produce proportionally more root than shoot biomass when N supply is limited[5,17]. In other words, when N is added to soils with low native N availability, plants will proportionally grow more shoots. This was the case in this study where allocation of biomass to leaves and branches was increased and to roots decreased by N and P applications (Fig. 2). Li et al.[5] hypothesized that seedlings that allocate more biomass to roots than aboveground parts may be better suited to a low N supply condition. Genotypes may have their own strategies in coping with the changing environment. Data reported in Nelson et al.[15] showed a trend different from the one we observed; in their case N fertilization increased biomass allocation to wood

(stem) rather than to foliage. This difference may be partly because we used 2-year-old seedlings and Nelson et al.[15] used 9-year-old plantations.

F10023 responded to N and P applications by investing proportionally more photosynthates in leaf production as compared with F10022. Families that allocate relatively more dry matter to shoots may grow faster because they reinvest a greater proportion of the photosynthates to tissues that are responsible for photosynthesis[18]. Corresponding to the greater biomass allocation of F10023 to leaves was the smaller allocation to stem. Nelson et al.[15] also demonstrated differences in biomass allocation between the two half-sib seed sources they tested, and explained such a trend as a result of differences in efficiencies in converting photosynthates into woody biomass. However, no root data were obtained in their study and the effects of N application on biomass allocation between roots and shoots were unknown.

### Plant N Uptake and N Use Efficiency

N application was very effective in raising N concentrations in all the components studied (Fig. 3). Foliar %N was at severely deficient levels in the two no-N treatments, while the levels in the N100 and N100-P50 treatments were at close to optimum and optimum levels, respectively (Lee Allen, North Carolina State University, personal communication). Higher foliar N concentrations in F10022 than in F10023 did not help to explain the greater biomass production in F10023; however, data reported in Chang and Robison[14] showed that F10023 seedlings had greater crown volume and total leaf area per seedling. This illustrates that, under the conditions used in this experiment, greater leaf area production was more important to biomass production than foliar N concentration[19].

Although leaves only constitute between 25 and 38% of the total seedling biomass (Fig. 2), the majority of the N that was taken up by the seedlings was found in the leaves (Fig. 4). The next largest pool of N was stored in the roots (Fig. 4). The N content in all the studied components was increased by N application. These distribution and response patterns have practical implications for nutrient conservation and for promoting future growth. For example, Kuers and Steinbeck[20] showed that fertilized sweetgum trees have higher N contents and reabsorbed more N from senescing foliage than the controls, thus conserving more N for next growing season. N stored in the root system may also be an important source of N for the next growing season, particularly in the early growing season. Increases in foliar N content in this study resulted from both increases in foliar biomass and foliar N concentration, which was consistent with Nelson et al.[15] and Kuers and Steinbeck[20].

Significant family differences in N content in foliage and branch components were consistent with the biomass and N concentration data. Nelson et al.[15] found no difference in N contents in aboveground biomass between the two half-sibs they tested. Further comparison is not possible because they did not present foliage and stem N content data.

One of the interesting findings from this study was that P application not only increased foliar P concentration levels (data not shown) but also promoted the uptake of N in the various aboveground seedling components (Fig. 4). The promotion of N uptake by P application was particularly strong when N was also applied. This N $\times$ P interaction in N uptake may be one of the indirect mechanisms where sweetgum growth was further improved by P addition. The direct mechanism is the improved P nutrition resulting from P application.

Our second hypothesis stated that sweetgum families that are more responsive to N additions will also have greater nutrient use efficiencies. This hypothesis was only partially supported by the data from this study: NUE was higher ($p < 0.05$) in F10023 than in F10022 only when P was applied; otherwise there was no difference in NUE between the two half-sibs. Our data showed that improved soil N availability increased seedling growth rate by utilizing proportionately more N per unit of biomass produced. In other words, N application increased tree size growth and biomass accumulation but decreased the overall NUE. This is consistent with analysis presented by Vitousek[21] and Reich and Schoettle[22] that NUE was higher in forests with lower N availability or plants with lower N content.

## CONCLUSIONS

In a pot experiment studied over one growing season, fertilization with N and P increased sweetgum total seedling biomass production and its allocation into foliage and, to a lesser extent, branches, while less was allocated into roots. Biomass production as well as its allocation patterns was different between the two half-sib families studied, indicating the potential usefulness of genotype selection for increasing growth responses to silvicultural treatments. Sweetgum seedlings were very responsive to N fertilization and, to a lesser degree, to P application. P application not only improved sweetgum P nutrition (direct effect) but also increased the uptake of N from the growth media resulting in greater yield increases (indirect effect).

The majority of N taken up by seedlings was stored in foliage and roots. This type of nutrient distribution pattern has practical implications: N contained in the leaves is required for photosynthesis and N content in roots may contribute part of the N required for growth during the early growing season when there are no leaves on the seedlings. At least in the short term, NUE was reduced by N application and proportionally greater amounts of nutrients are needed to achieve higher biomass yield.

## ACKNOWLEDGMENTS

Funding from the member companies of the HRC of the North Carolina State University is greatly appreciated. We thank HRC staff for assistance in setting up the experiment and in sample collection and preparation. The North Carolina Division of Agronomy kindly performed chemical analysis of the plant samples. We thank Drs. Phillip G. Comeau, Michael G. Shelton, and Karen Kuers for reviewing earlier versions of the manuscript.

| | | |
|---|---|---|
| Received: | July | 16, 2001 |
| Revised: | September | 17, 2001 |
| Accepted: | October | 5, 2001 |
| Published: | October | 23, 2001 |

## BIOSKETCH

**Scott Chang** is an assistant professor in forest soils in the Department of Renewable Resources, University of Alberta. Before joining the University of Alberta, he held academic positions at Lincoln University in New Zealand and North Carolina State University. He received his Ph.D. degree in 1996 from the University of British Columbia. Dr. Chang's research interests are in the general areas of forest soils and tree nutrition. Currently he is looking at the effect of soil compaction and organic matter removal on tree ecophysiology and soil processes. He is also studying the potential of using forest fertilization as a tool to increase the productivity of boreal forests and is interested in the complex relationships among forest structure, soil temperature and soil nutrient dynamics, and their implications for site productivity and global change.
